# Supplementary material for: Influence of Atherosclerosis-Associated Risk Factors on Expression of Endothelin Receptors in Advanced Atherosclerosis
Source: Int J Mol Sci. 2025 Mar 5;26(5):2310. doi: 10.3390/ijms26052310 (PMC11899768; doi:10.3390/ijms26052310)
Supplement: Supplementary file 1 [file ijms-26-02310-s001.zip › ijms-3456405-supplementary.pdf]

Supplementary Materials

Table S1 Precellys homogenisation programme

rounds per minute	time	pause
5500	30 seconds	60 seconds

Table S2 Composition of qRT-PCR reaction mix.

Amount	Component
10 µl	2X KAPA Mastermix
200 nm	Primer (see table S1)
0,4 µl	50X KAPA RT-Mix or H2O for NTC
20 pg	Template-RNA
Amount	Component

Samples were filled up with HPLC- H_2O up to a final volume of 50 µl

Table S3 qRT primer details

targeted human RNA	Primer name	Order-ID
Endothelin-1	Hs_EDN1_1_SG	QT00088235
Endothelin receptor type A	Hs_EDNRA_1_SG	QT00030156
Endothelin receptor type B	Hs_EDNRB_1_SG	QT00014343
Matrix Metalloproteinase 2	Hs_MMP2_1_SG	QT00088396
Smooth muscle actin	Hs_ACTA2_1_SG	QT00088102
GAPDH	Hs_GAPDH_1_SG	QT00079247

Table S4 qRT-PCR programme

Step	Temperature	Time
Reverse Transcription	42 °	5 minutes
Enyme activation	95 °C	3 minutes
Denaturation	95 °C	10 seconds
Anealing	60 °C	20 seconds
Extension	72°C	20 seconds

40 PCR cycles of denaturation, annealing and extension were used in total followed by a melting curve (95 °C, 15 seconds; 60 °C, 15 seconds; 95 °C, 15 seconds).

Table S5 Kolmogorov-Smirnow test results

gene	Statistik p-value
ETA-R	.066
ETB-R	.055
ET1	.073
ACTA2	.076
MMP2	.063

p-value above 0,05 indicates Gaussian distribution.

Table S6 Cohort data showing the complete data set of each proband

No.	type	symptoms	NASCET score	age	gender	nicotine abuse	height (m)	weight (kg)	hypertension	diabetes mellitus	adipositas	renal disease	myocardial infarction	stroke	cancer	hepatic disease	medication status	family medical history
1	control	na	na	33	f	y	1,63	87	n	n	n	n	n	n	n	n	n	n
2	control	na	na	24	m	y	1,85	85	n	n	n	n	n	n	n	n	n	y
3	control	na	na	50	f	n	1,72	76	n	n	n	n	n	n	n	n	n	n
4	control	na	na	37	m	y	1,84	79	n	n	n	n	n	n	n	n	n	n
5	control	na	na	51	f	n	1,65	72	n	n	n	n	n	n	n	n	n	n
6	control	na	na	74	f	n	1,65	70	n	n	n	n	n	n	n	n	n	n
7	control	na	na	46	f	n	1,65	70	n	n	n	n	n	n	n	n	n	n
8	control	na	na	69	m	n	1,74	90	n	y	y	n	n	n	n	n	n	n
9	control	na	na	63	f	n	1,74	65	n	n	n	n	n	n	n	n	n	n
10	control	na	na	32	f	n	1,65	75	n	n	n	n	n	n	n	n	n	n
11	control	na	na	64	f	n	1,66	70	n	n	n	n	n	n	n	n	n	n

12	control	na	na	59	m	n	1,73	84	n	n	n	n	n	n	n	n	n	n
13	control	y	na	30	na	na	1,77	55	n	n	n	n	n	n	n	n	y	n
14	control	y	na	42	f	na	1,64	103	na	y	y	y	na	na	na	n	y	y
15	control	na	na	46	f	y	1,61	68	n	n	n	n	n	n	n	n	n	n
16	control	na	na	29	m	n	1,83	83	n	n	n	n	n	n	n	n	n	n
17	control	na	na	55	f	n	1,65	59	n	n	n	n	n	n	n	n	n	n
18	control	na	na	27	f	n	1,68	72	n	n	n	n	n	n	n	n	n	n
19	control	na	na	28	f	n	1,64	65	n	n	n	n	n	n	n	n	n	n
20	control	na	na	69	f	n	1,66	59	y	n	n	n	n	n	n	n	y	n
21	control	na	na	40	f	n	1,9	100	y	y	y	n	n	n	n	n	y	n
22	control	na	na	77	m	n	1,77	84	y	n	n	n	n	n	n	n	y	n
23	control	na	na	34	f	y	1,65	68	n	n	n	n	n	n	n	n	n	n
24	control	na	na	63	m	n	1,76	86	n	n	n	n	n	n	n	n	n	n
25	control	na	na	69	f	n	1,6	80	y	n	y	n	n	n	n	n	y	n
26	control	na	na	62	m	y	1,76	140	n	n	y	n	n	n	n	n	y	n
27	carotid	y	80	85	f	n	1,65	70	y	n	n	n	n	y	n	n	y	n
28	carotid	n	80	76	m	n	1,68	81	y	n	y	n	y	n	n	n	y	n
29	carotid	y	50	80	m	n	1,67	70	y	n	n	y	n	y	n	n	y	n
30	carotid	n	na	58	f	y	1,64	56	y	n	n	n	n	n	na	n	y	n
31	carotid	y	na	66	m	y	1,78	90	na	y	na	na	na	na	na	na	na	n
32	carotid	n	80	66	m	n	1,65	84	y	n	n	n	y	n	n	n	y	n
33	carotid	n	90	68	m	n	1,72	90	y	y	n	y	n	y	n	n	y	n

34	carot id	y	na	na	m	y	1,83	90	y	n	n	y	n	n	na	n	y	n
35	carot id	y	80	72	f	n	1,7	66	y	n	n	n	n	n	n	n	y	n
36	carot id	y	80	86	m	n	1,75	84	y	y	n	y	y	y	y	n	y	n
37	carot id	y	80	75	m	n	1,75	81	y	n	n	n	n	y	n	n	y	n
38	carot id	y	na	61	m	y	1,7	57	n	n	n	na	na	na	na	na	y	y
39	carot id	n	80	79	m	n	1,84	87	y	n	n	n	n	n	n	n	y	y
40	carot id	y	na	68	f	n	1,68	70	y	n	n	n	n	y	na	n	y	y
41	carot id	y	90	72	m	n	1,83	95	y	y	n	n	y	y	n	n	y	n
42	carot id	y	na	58	f	y	1,67	65	y	n	n	na	n	n	na	na	y	y
43	carot id	n	80	78	m	n	1,78	85	y	n	n	n	n	n	n	n	y	n
44	carot id	n	90	68	m	n	1,8	85	y	n	n	n	n	n	n	n	y	n
45	carot id	y	80	72	f	y	1,57	61	y	n	n	n	n	y	y	n	y	n
46	carot id	n	80	58	m	n	1,85	90	y	n	n	n	n	n	n	n	y	n
47	carot id	n	80	62	f	y	1,71	83	y	n	n	n	n	n	n	n	y	n
48	carot id	y	70	76	m	y	1,8	84	y	n	n	n	n	n	n	n	y	y
49	carot id	y	na	56	f	y	1,62	64	na	n	n	n	n	n	na	n	y	y
50	carot id	y	60	61	m	n	1,83	94	y	n	y	n	n	y	n	n	y	y
51	carot id	n	80	56	f	n	1,65	58	y	n	n	n	n	n	y	n	y	n
52	carot id	n	80	68	f	n	1,52	63	y	n	n	n	n	n	y	n	y	n
53	carot id	n	80	51	m	n	1,83	100	y	n	y	n	y	n	n	n	y	n
54	carot id	y	70	63	m	y	1,83	110	y	n	y	n	n	y	n	n	y	y
55	carot id	n	70	71	m	y	1,65	70	y	n	n	n	y	n	n	n	y	y

56	carot id	n	80	59	f	y	1,69	54	n	n	n	n	n	n	n	n	y	y
57	carot id	y	70	84	f	n	1,66	63	y	n	n	n	n	n	n	n	y	n
58	carot id	y	80	80	m	n	1,7	71	y	y	n	n	y	y	n	n	y	n
59	carot id	n	80	69	m	n	1,91	118	y	y	y	n	n	n	n	n	y	n
60	carot id	y	80	52	m	n	1,84	110	n	n	y	n	n	n	n	n	y	n
61	carot id	y	80	86	m	n	1,84	97	y	y	y	n	n	y	n	n	y	n
62	carot id	na	80	70	m	y	1,78	70	y	n	n	n	n	n	n	n	y	n
63	carot id	n	70	65	m	n	1,85	90	y	n	n	n	n	n	n	n	y	y
64	carot id	n	80	66	m	n	1,74	89	y	n	y	n	y	n	n	n	y	y
65	carot id	y	80	71	m	n	1,76	113	y	n	y	y	y	y	n	n	y	y
66	carot id	n	90	71	m	n	1,76	76	y	y	n	n	y	n	n	n	y	n
67	carot id	y	80	78	m	n	1,8	90	y	y	n	n	n	y	n	n	y	y
68	carot id	n	90	74	m	n	1,77	85	y	n	n	n	n	n	n	n	y	y
69	carot id	y	80	77	m	n	1,75	100	y	y	n	n	y	n	n	n	y	n
70	carot id	n	60	68	m	y	1,78	75	y	n	n	y	y	y	n	n	y	n
71	carot id	y	60	78	f	y	1,73	64	y	n	n	n	n	n	n	n	y	n
72	carot id	y	70	59	f	n	1,68	84	y	n	n	n	n	y	n	n	y	y
73	carot id	n	90	78	m	n	1,72	72	y	n	n	n	n	y	n	n	y	n
74	carot id	y	80	50	m	n	1,8	90	y	n	n	n	n	y	n	n	y	n
75	carot id	n	80	63	f	n	1,68	62	y	n	n	n	n	y	n	n	y	n
76	carot id	y	70	60	f	y	1,7	63	y	n	n	n	n	n	n	n	y	n
77	carot id	n	90	74	f	y	1,8	78	n	n	n	n	n	n	n	n	y	y

78	carotid	n	90	63	m	y	1,8	90	y	n	n	n	n	n	n	n	y	n
79	carotid	y	70	52	m	n	1,73	65	n	n	n	n	n	y	n	y	y	y
80	carotid	y	80	59	m	y	1,81	85	y	n	n	n	n	n	n	n	y	n
81	carotid	y	90	83	m	n	1,79	82	y	n	n	n	n	n	n	n	y	y
82	carotid	n	80	89	m	n	1,78	95	y	n	y	n	n	n	n	n	y	n
83	femoral	y	na	68	f	n	1,52	59	y	n	n	n	n	y	y	n	y	n
84	femoral	na	na	71	m	n	1,7	80	y	n	y	n	n	n	n	n	y	n
85	femoral	na	na	63	m	y	1,66	86	y	n	n	n	n	n	n	n	y	n
86	femoral	na	na	72	m	n	1,8	80	y	n	n	n	n	n	n	n	y	n
87	femoral	na	na	58	m	n	1,76	86	y	n	y	n	n	n	y	n	y	y
88	femoral	na	na	76	m	n	1,8	76	y	y	y	n	y	n	n	n	y	n
89	femoral	y	na	77	f	n	1,62	82	y	y	y	y	n	n	na	n	y	n
90	femoral	y	na	62	m	y	1,73	77	y	n	n	n	n	n	na	n	y	y
91	femoral	na	na	68	m	y	1,8	86	y	n	y	n	n	n	n	n	y	n
92	femoral	na	na	76	m	y	1,68	82	y	y	y	y	y	n	n	n	y	n
93	femoral	n	80	74	m	n	1,82	89	y	y	n	n	n	n	n	n	y	y
94	femoral	y	80	79	m	n	1,69	90	y	n	y	n	y	n	y	n	y	n
95	femoral	y	na	54	m	n	1,64	85	y	y	y	n	n	n	n	n	y	n
96	femoral	y	na	57	m	n	1,8	90	y	y	n	n	y	n	n	n	y	y
97	femoral	y	na	70	m	y	1,8	82	y	y	n	n	y	n	n	n	y	y
98	femoral	y	na	60	m	y	1,84	86	y	n	n	n	n	n	n	n	y	y

y= yes, n = no, na = no answer, m = male, f = female

Table S7 Details of regression analysis depicted in Figure 3

risk factor	gene	regression coefficient	95% CI
**age**	*ETA-R*	-0,011	-0,019--0,003
	ETB-R	-0,003	-0,01-0,005
	E1	0,001	-0,008-0,01
	ACTA2	-0.013	-0,022--0,004
	MMP2	-0,005	-0,013-0,004
**gender**	*ETA-R*	-0,167	-0,368-0,033
	ETB-R	-0,284	-0,479--0,0088
	ET1	0,002	-0,236-0,24
	ACTA2	-0,095	-0,327-0,138
	MMP2	0,101	-0,114-0,316
**smoking**	*ETA-R*	0,026	0,167-0,219
	ETB-R	-0,077	-0,265-0,111
	ET1	-0,043	-0,272-0,186
	ACTA2	-0,190	-0,413--0,032
	MMP2	-0,029	-0,235-0,177
**BMI**	*ETA-R*	0,031	0,007-0,055
	ETB-R	0,001	-0,023-0,024
	ET1	-0,001	-0,029-0,028
	ACTA2	0,012	-0,016-0,04
	MMP2	-0,012	-0,037-0,014
**hypertension**	*ETA-R*	-0,284	-0,533--0,036
	ETB-R	-0,39	-0,636--0,145
	ET1	-0,233	-0,532--0,066
	ACTA2	-0,324	-0,615--0,032
	MMP2	0,142	-0,127-0,412
**diabetes mellitus**	*ETA-R*	0,095	-0,146-,336
	ETB-R	0,157	-0,07-0,384
	ET1	0,228	-0,05-0,505

ACTA2	0,174	-0,096-0,445
MMP2	0,111	-0,139-0,360

CI = confidence intervall

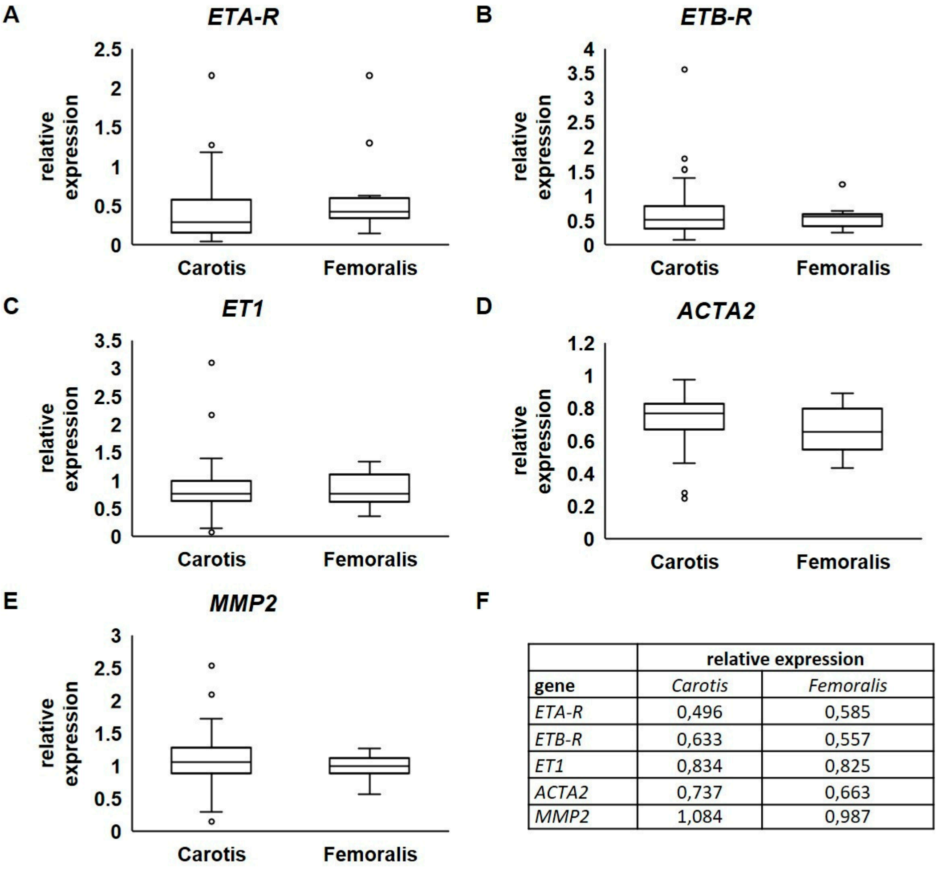

	relative expression	
**gene**	*Carotis*	*Femoralis*
ETA-R	0,496	0,585
ETB-R	0,633	0,557
ET1	0,834	0,825
ACTA2	0,737	0,663
MMP2	1,084	0,987

Figure S1. Comparison of gene expression differences of the carotid und femoral specimen. A-F There are no significant differences in gene expression between the carotid und femoral specimen for all genes examined.

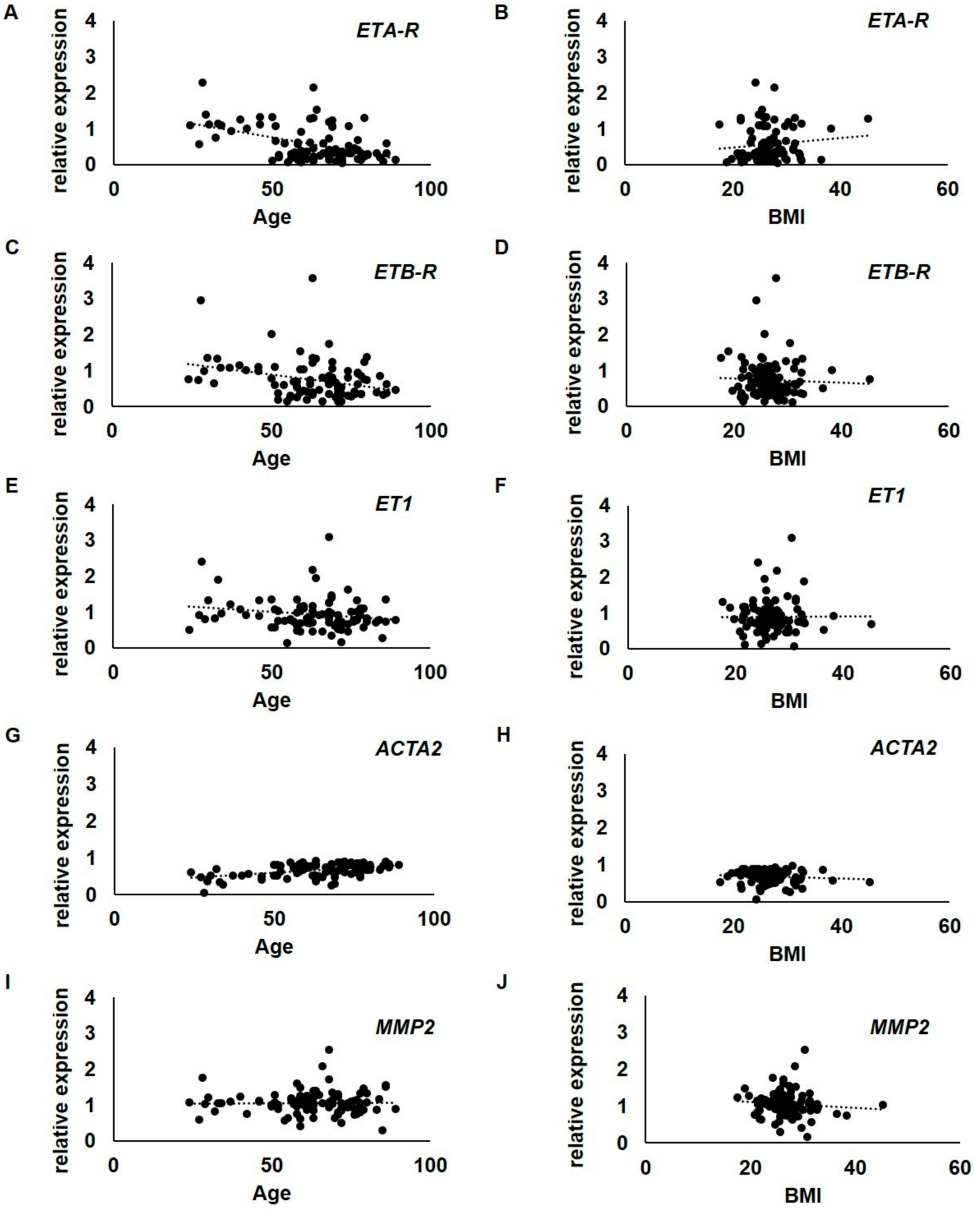

Figure S2. Scatter plots of individual expression against the two non-dichotomous variables, age and BMI, indicate an approximately linear relationship.
